# Compliance with Ministry of Health Regulations among Israeli Nurses during the COVID-19 Pandemic: The Mediating Role of Risk Perception

**DOI:** 10.3390/healthcare11040601

**Published:** 2023-02-17

**Authors:** Shiran Bord, Shosh Shahrabani, Hagar Baruch, Hanna Admi

**Affiliations:** 1 Department of Health Systems Management, The Max Stern Yezreel Valley College, Yezreel Valley 1930600, Israel; 2 Department of Economics, The Max Stern Yezreel Valley College, Yezreel Valley 1930600, Israel; 3 Ramban Hospital, Haifa 3109601, Israel; 4 Department of Nursing, The Max Stern Yezreel Valley College, Yezreel Valley 1930600, Israel

**Keywords:** risk perception, health belief model, emotions, nurses

## Abstract

The COVID-19 pandemic has created a sustained state of emergency, causing uncertainty and risk taking. Israeli nurses were required to follow new regulations and safety measures issued by the Israeli Ministry of Health (MOH). This study aimed to examine nurses’ compliance with MOH regulations and its association with their risk and threat perceptions and their positive and negative emotions. A cross-sectional online survey was conducted among 346 Israeli nurses. The study model was examined with path analysis. Most nurses reported complying with MOH regulations either fully (49%) or very often (30%). Negative emotions were positively associated with perceptions of both threat and risk, yet only risk perception was positively associated with nurses’ compliance. A significant mediated relationship was found between negative emotions and nurses’ compliance, with the possible mediator being risk perception. Hence, higher negative emotions were associated with a greater risk perception, which was associated with higher compliance. Health systems leaders must strategize to deal with the wave-like character of the pandemic. Solutions to nursing teams’ negative emotions must be provided to keep the balance between feelings of complacency and a situation of high-level, intense negative feelings, which might lead to abstention, burnout, or emotional injury.

## 1. Introduction

The coronavirus disease (COVID-19) was declared a global pandemic by the World Health Organization (WHO) in March 2020 [1], causing major changes in health systems worldwide. To prevent the public and medical teams from contracting the virus, the Israeli Ministry of Health (MOH) followed the WHO’s guidelines, focusing on instructions aimed at minimizing contact and maintaining optimal hygiene and physical distancing. Medical teams in general, and nurses in particular, are on the frontlines of the pandemic, necessitating the making of clinical and behavioral decisions on a daily basis. Their compliance with the Ministry of Health regulations and the factors associated with them are of the utmost importance in preserving and promoting their own and their patients’ health. The COVID-19 pandemic has forced medical teams into a sustained state of emergency, and with it, much uncertainty and risk taking. Managing teams and human resources during a crisis of this kind presents a challenge, both in terms of personal coping and in the health care system’s need to support the teams dealing with the crisis [2].

A meta-analysis conducted during the first wave of the COVID-19 pandemic suggested that a considerable proportion of healthcare workers (HCWs) experienced mood and sleep disturbances during the pandemic [3]. Another study conducted during the pandemic in New York City found that a high percent of the nurses experienced COVID-19-related psychological distress [4]. Furthermore, a recent study conducted among Australian frontline healthcare workers [5] has found that more than 70% of respondents report that the pandemic has affected their relationships with their friends, family members and colleagues, as well as having mental difficulties, including anxiety and depression.

Studies have shown that negative emotions, such as fear and anxiety, are associated with an increased risk perception, which in turn influences the decision-making process in general, and among medical teams in particular [6,7]. A recent study conducted in China during the pandemic has found a higher level of perceived risk in relation to COVID-19, as compared to other potential health threats. Furthermore, this study indicates that risk perception is positively related to depressive states [8]. The literature suggests that emotions constitute potentially harmful or beneficial drivers of decision making and may influence judgment and choice [6]. Therefore, emotions are of great importance when attempting to understand and explain nurses’ decision making during the COVID-19 pandemic.

Several researchers have taken a valence-based approach to emotions, contrasting the influence of negative emotions on decision making with those of positive emotions. For example, according to this approach, Johnson and Tversky [9] found that participants induced to feel negative emotions consistently made more pessimistic estimates regarding frequencies of death than participants induced to feel positive emotions. In line with this framework, several empirical studies show that in the context of major events, such as wars, higher levels of negative emotions predict pessimistic risk assessments [10,11,12,13]. Moreover, Maner and Gerend [14] indicate that negative emotions, such as fear, reflect basic motivational orientations related to avoidance; therefore, fear is expected to be associated with risk perception facilitating risk avoidance.

Slovic [15] has identified two major predictors of people’ risk perceptions: (1) perceived lack of control in the situation, and (2) the novelty of the risk, i.e., the extent to which the risk is new or unknown. The COVID-19 pandemic has exposed nurses to an unknown new virus. Considering the scientific literature, it is likely that nurses’ risk perceptions may influence both their decision making and their compliance during this crisis.

Risk perceptions may also be associated with the perceived threat of the disease. The Health Belief Model [HBM] [16] relates to a specific aspect of risk perception, defined as the individual ‘perceived threat’. The HBM mentions the perceived susceptibility of the individual and perceived severity of the disease, describing to what extent people believe they are susceptible to the disease or its consequences, and the levels of severity they associate with them. According to this model, the combination of both perceived severity and perceived susceptibility defines the individual’s perceived threat regarding the disease.

According to the HBM, motivation for health behaviors and compliance is greater when perceived sensitivity to disease and perceived illness severity are greater, and the higher the ‘threat perception’, the higher the perceived benefits of preventive actions, and the lower the level of barriers to performing preventive actions [17,18,19]. This is further supported by a study conducted by Ganz and his colleagues [20], which showed that nurses’ perceptions of disease threat and threat types were (positively or negatively) associated with their willingness to work under these conditions.

Foye and her colleagues [21] have found that nurses report that compliance with all regulations during the pandemic may be challenging, and in many cases impossible. Another study [22] conducted in Switzerland has found that only 8% of healthcare workers always succeeded in keeping the required distance from others; however, most of them complied with the regulation concerning washing hands and staying at home when feeling sick. The results of a recent study conducted among Israeli physicians [23] shows that only 61% of the respondents reported full compliance with COVID-19-related MOH guidelines.

The current study’s aim was to examine nurses’ compliance with MOH regulations and its association with the nurses’ risk and threat perceptions, and their positive and negative emotions, during the COVID-19 pandemic in Israel. 

Our main hypothesis was that nurses’ emotions during the epidemic are associated with their risk and threat perceptions, which are, in turn, associated with their compliance. Hence, consistent with valence theory [9], we hypothesized that higher levels of negative emotions among nurses would be associated with higher levels of perceived risk and, based on the HBM [16], nurses who perceived a greater threat of COVID-19 would tend to be more compliant with MOH guidelines and would take more precautions in their decision-making processes.

## 2. Materials and Methods

The current study is a cross-sectional survey among Israeli nurses. Data were collected between 29 March and 10 May 2020, via an online questionnaire delivered by a professional survey company. 

### 2.1. Participants Selection and Inclusion Criteria

Participants were recruited, as mentioned above, by a professional survey company. The survey company recruited respondents by approaching professional nurses’ associations and distributing the online link in Israeli hospitals using snowball sampling. The company approached head nurses in five big hospitals in Israel: Soroka Medical Center, Hadassa University Medical Center, Kaplan Medical Center, Shaare Zedek Medical Center, and Sheba Tel Hashomer, and asked them to distribute the study questionnaire among the nursing staff. Furthermore, the company approached nurses working in community settings, such as Clalit and Macabi health services, and asked them to distribute the study questionnaire in their workplace. In order to increase responsiveness and reduce possible selectivity bias, several reminders were sent to the respondents by the head nurses (since minimizing the nonresponse rate may reduce selection bias).

In addition to the professional survey company’s efforts, two of the researchers (H.A. and H.B.) are senior nurses, one of them (H.A.) a former head nurse of Rambam hospital, and the other (H.B.) is the Deputy Director of Nursing at the same hospital. Moreover, one of the researchers (H.A.) is a professor of Nursing and acting head of MA studies in Nursing at the Max Stern Yezreel Valley college. After obtaining ethical approval from Rambam Medical Center and The Max Stern Yezreel Valley College, the researchers further distributed the study questionnaire to nurses at Rambam Medical Center and 4th year nursing students at Yezreel Valley College.

The sample size was calculated to represent the target population. According to the calculation, 321 respondents were required (with 95% confidence level and a sampling error of ±5%). All nurses currently working in the profession were invited to answer the questionnaire. Moreover, during the pandemic in Israel, 4th year nursing students were recruited to work in hospitals and were, therefore, also included in the current study sample. A total of 346 nurses were included in the final sample.

### 2.2. Measures and Variables Description

The study questionnaire included 78 items and was written in Hebrew. To measure the dependent variable, the researchers used three items based on the Israeli MOH COVID-19 guidelines. The questions measuring the study’s independent variables were based upon existing valid and reliable questionnaires (as detailed below), translated from English to Hebrew (and back-translated), and adjusted to the current study’s aims and target population. All the researchers went over the questionnaire, and only after it was corrected and finalized (test–retest) was it sent to the survey company for distribution.

The dependent variable was the *nurses’ compliance with MOH regulations* (compliance), reflecting their decision-making process regarding how to behave during the pandemic. To measure nurses’ compliance, we used three items as detailed below (Cronbach’s alpha  =  0.69). The nurses were asked to indicate the extent to which they followed the following MOH regulations: ‘Being more than usually meticulous regarding hygiene rules at work’; ‘Being more than usually meticulous regarding hygiene rules at home’; and ‘Being more than usually meticulous regarding the following of COVID-19 regulations issued by the MOH’. The response options ranged from ‘not at all’ (1) to ‘Fully’ (7).

Four primary independent variables were used: negative and positive emotions, risk perception, and COVID-19 threat perception. In addition, sociodemographic and background characteristics were collected.

*Negative and positive emotional levels* were measured using the Positive and Negative Affect Schedule (PANAS) questionnaire [24]. We computed two variables using the respondents’ mean response to the question ‘How often have you felt these emotions during the last week?’: (1) Negative emotions—including stress, a bad mood, blame, anxiety, nervousness, fear, anger and frustration (Cronbach’s alpha  =  0.88); and (2) positive emotions—including strength, enthusiasm, pride, relaxation, activism and sense of mission (Cronbach’s alpha  = 0.78). The response options ranged from ‘not at all’ (1) to ‘felt very strongly’ (7).

*Threat perception* was measured based on the HBM subscale of the perceived threat [17,25,26]. Seven items were used to measure the nurses’ perceived risk of COVID-19 to self and significant others (Cronbach’s alpha  = 0 .79): ‘My chances of getting COVID-19 are high’; ‘The thought of getting COVID-19 scares me’; ‘COVID-19 can be a serious disease and can cause medical complications and even death’; ‘I worry a lot about getting COVID-19′; ‘If I get COVID-19 it would make my family nervous and scared’; ‘If I get COVID-19 it will harm my functioning’; and ‘Working with multiple people each day increases my chances of getting COVID-19 and infecting my family’. The response options ranged from ‘strongly disagree’ (1) to ‘strongly agree’ (7).

*Nurses’ risk perception* was measured based on the Domain-Specific Risk-Taking (DOSPERT) scale [27]. We computed this variable using the respondents’ mean response to the question ‘How dangerous are the following actions, in your opinion?’ (Cronbach’s alpha  = 0.83), using nine actions: treating a patient suspected with COVID-19 without protective equipment; treating a COVID-19 patient without protective equipment; working in a hospital; working in the community (community clinics); touching public surfaces without gloves; not washing hands after touching public surfaces; meeting family members; using public transportation; and shopping for food and drugs. The response options ranged from ‘not dangerous at all’ (1) to ‘extremely dangerous’ (7).

### 2.3. Data Analysis

Data were analyzed using SPSS ver. 26. Internal consistencies were examined, and variables were composed with item means. The variable of compliance was negatively skewed and, thus, exponentially transformed. The study variables were described with means and standard deviations, and Pearson correlations were calculated between them. Independent t-tests were calculated for the study variables, by high/low compliance with MOH guidelines. Pearson correlations and independent t-tests were calculated between the study variables and the demographic characteristics, to identify background variables that need to be controlled for. The study model was examined with path analysis, using AMOS ver. 26. Chi square, NFI, NNFI, CFI, and RMSEA were used as measures of fit. Age, ethnicity, and religiosity level were controlled for. Control variables were allowed to correlate among themselves, and so were the two independent variables and the two mediators. Mediation was examined within the path analysis, with bootstrapping of 2000 samples and a bias-corrected 95% confidence interval. All variables were standardized.

### 2.4. Ethical Considerations

The study protocol was approved by The Yezreel Valley College ethics committee board (approval ref. 2020-67 YVC EMEK). Ethical guidelines have been met, including adherence to Israeli legal requirements. The survey questionnaire was anonymous, and no personal information was provided.

## 3. Results

The participants were 346 nurses between the ages of 21 and 71 (mean = 41.49 years, SD = 10.45), having practiced nursing for up to 48 years (mean = 16.27 years, SD = 11.62) (see Table 1).

Most were registered nurses (87%), working in hospitals (80%), and others were nursing students (in their 4th year, recruited to work in hospitals during the pandemic) and nurses working in various community services or health-related facilities. They were mostly female (84%), Jewish (75%), Israeli-born (70%), married or in a meaningful relationship (74%), and in an average or above-average economic status (73%). Over half were secular (57%), and others were partly religious (24%) or religious (19%).

Nurses were asked about the extent to which they complied with hygiene regulations and the regulations issued by the MOH pertaining to the COVID-19 outbreak. Most nurses responded that they complied with hygiene regulations at work fully (61%) or very often (25%). They tended to comply with these regulations less closely at home (fully 38%, very often-32%). Most nurses, however, reported complying with the general MOH regulations either fully (49%) or very often (30%).

Indeed, as shown in Table 2, mean compliance was high (M = 6.16, range 1–7). Means for threat and risk perceptions were moderate–high, the mean for positive emotions was moderate, and the mean for negative emotions was moderate–low. Positive correlations were found between the compliance level and perceptions of both threat and risk; the latter, which were positively interrelated, were positively associated with negative emotions and negatively associated with positive emotions. Negative and positive emotions were, as expected, negatively interrelated.

The most common positive emotions were sense of mission (63%), feeling strong (55.5%), and feeling proud (54.6%), while the most common negative emotions reported were frustration (31.8%), stress (30%), and a bad mood (28.4%).

For a better understanding of the nurses’ compliance with MOH regulations, the nurses were divided into two groups: those complying with the regulations closely (mean compliance over 6, *n* = 210) versus those complying with them less closely (mean compliance up to 5.99, *n* = 136). The comparison, as shown in Table 3, reveals that higher compliance was associated with greater threat and risk perceptions, but no differences were found in negative or positive emotions between the two groups.

Pearson correlations and a series of t-tests were calculated between the study variables and the demographic characteristics. Age was negatively associated with negative emotions (r = −0.23, *p* < 0.001) and with risk perception (r = −0.13, *p* = −0.016). Hence, older nurses reported lower negative emotional levels and lower risk perceptions. Seniority in nursing was, as expected, highly associated with age (r = −0.88, *p* < 0.001).

Nurses’ compliance was higher for non-Jewish respondents (M = 6.31, SD = 0.81) than for Jewish ones (M = 6.11, SD = 0.88) (t(339) = 2.33, *p* = 0.021) and higher for nonsecular nurses (M = 6.29, SD = 0.81) as compared to secular ones (M = 6.06, SD = 0.90) (t(339) = 2.54, *p* = 0.012).

Furthermore, negative emotions were higher for non-Jewish nurses (M = 3.14, SD = 1.28) as compared to Jewish nurses (M = 2.79, SD = 1.20) (t(339) = 2.31, *p* = 0.022). Likewise, risk perception was higher for non-Jewish respondents (M = 5.69, SD = 0.85) than for Jewish ones (M = 5.38, SD = 0.77) (t(339) = 3.22, *p* = 0.001).

In addition, threat perception was higher among nonsecular nurses (M = 5.32, SD = 1.00) as compared to secular ones (M = 5.02, SD = 1.11) (t(339) = 2.58, *p* = 0.010); similarly, risk perception was higher for nonsecular nurses (M = 5.57, SD = 0.80) than for secular ones (M = 5.37, SD = 0.79) (t(339) = 2.29, *p* = 0.023).

Ethnicity and religiosity were weakly correlated (Phi = 0.13, *p* = 0.014). Other differences were not significant; thus, the study model was examined while controlling for age, ethnicity (0—non-Jewish, 1—Jewish), and religiosity (0—nonsecular, 1—secular).

The study model was examined with path analysis, using AMOS ver. 26. Age, ethnicity, and religiosity were controlled for. Control variables were allowed to correlate among themselves, and so were the two independent variables and the two mediators. Mediation was examined within the path analysis, with a bootstrapping of 2000 samples and a bias-corrected 95% confidence interval. Specific mediation effects were interpreted with the Monte Carlo method for assessing mediation [28,29], with a bootstrapping of 2000 samples and a 95% confidence interval. All variables were standardized. For the sake of clarity, only significant paths are shown in the figure, and others are presented in the table.

The results show that negative emotions were positively associated with perceptions of both threat and risk, yet only risk perception was positively associated with nurses’ compliance. Positive emotions were negatively associated with negative emotions, yet were unrelated to threat or risk perceptions, nor to nurses’ compliance.

**Figure 1:**
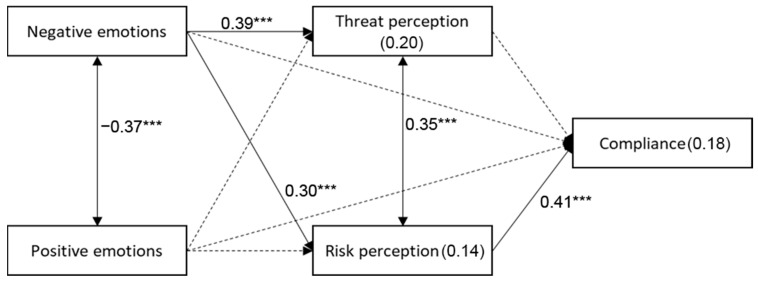
Path analysis for nurses’ behavior, threat and risk perceptions, negative and positive emotions. The model was found to fit the data: χ^2^(11) = 12.18, *p* = 0.350, NFI = 0.964, NNFI = 0.990, CFI = 0.996, RMSEA = 0.018. Table 4 and Figure 1 present the results of the path analysis. Note. R2-values within rectangles. Β-standardized regression coefficients—values above one-way arrows. R-correlations alongside two-way arrows. *** *p* < 0.001.

A mediated relationship was found to be significant in the path model between negative emotions and nurses’ behavior (standardized indirect effect = 0.157, SE = 0.031, 95% CI = 0.100, 0.211, *p* = 0.016). As shown in Table 4 and Figure 1, the possible mediator is risk perception. Its interpretation with the Monte Carlo method for assessing mediation [12,16], using a bootstrapping of 2000 samples and a 95% confidence interval, revealed its significance (95% CI = 0.075, 0.186). Thus, higher negative emotions were associated with greater risk perception, which was then associated with higher compliance with MOH regulations.

## 4. Discussion

The current study was designed to examine Israeli nurses’ compliance with MOH regulations and its association with their risk and threat perceptions and their positive and negative emotions during the first wave of the COVID-19 pandemic in Israel. Overall, compliance was high. This is no surprise, considering the research population and the scientific literature [21]; however, full compliance may be a challenge considering the new COVID-19 regulations [22].

Nurses’ compliance was higher for non-Jewish respondents than for Jewish ones, and higher for nonsecular nurses as compared to secular ones. These findings are very interesting since many other studies conducted during the COVID-19 crisis in Israel have shown the opposite (lower compliance among ultra-orthodox and Arab populations) [18,19]. A possible explanation for this might be the unique characteristics of the research population (nurses and nursing students only) as compared to other studies who examined compliance among the general population. The study’s findings affirm the research hypothesis, demonstrating that risk and threat perceptions and nurses’ emotions have an important role in their decision-making processes and their compliance level with MOH regulations during the pandemic. As mentioned above, nurses’ compliance with regulations was mostly high, with work-related higher than home-related regulations. The nurses’ risk and threat perceptions regarding the pandemic were moderate–high, but the level of negative emotions reported was mostly moderate–low. In the current study, older nurses with seniority in nursing reported lower negative emotional levels and lower risk perceptions. Similar findings were found in a similar study conducted among Israeli physicians [23], showing that older age and seniority among medical and nursing teams may be associated with regulation of the emotional response.

The level of positive emotions reported by the nurses was moderate, with the central positive emotions experienced during the pandemic being a sense of mission, feeling strong, and feeling proud. The strongest negative emotions reported were frustration, pressure, and a bad mood. Compliance with MOH regulations was found to be higher among nurses reporting higher risk and threat perceptions. These findings match the theoretical background discussed above.

The present study did not find a direct association between threat perception and compliance, as demonstrated previously [23,30,31]. However, a positive association was found between risk and threat perceptions, so risk perception in the present study constitutes a mediating variable between threat perception and compliance with the new regulations. Beyond what is reported in the literature regarding the association between risk and threat perceptions, when discussing the COVID-19 pandemic, it is reasonable to assume that pandemic-related threat perception will be translated in practice into risk perception both at work and at home, and this risk perception is directly connected to compliance with MOH regulations. According to Slovic [15], a high level of risk perception can be dependent on sensations of lack of control, as well as the risk’s innovativeness. There is no question that the COVID-19 crisis forced nurses to deal with a new, unfamiliar threat, as well as sensations of lack of control, mostly in the pandemic’s initial stages.

The model of the present study demonstrates that nurses’ risk perceptions serve as a mediating factor between negative emotions and compliance with regulations. In other words, nurses who reported stronger negative emotions, such as frustration, fear, and pressure, perceived the threat as higher, and thus complied more with MOH regulations. This finding matches the assumption behind the valence theory (Johnson & Tversky, 1983), according to which negative emotions will lead to a more pessimistic assessment of the situation and a higher risk perception. This is an extremely significant finding, as it points at the fact that higher negative emotions may, up to a point, lead to higher compliance; however, the literature shows that beyond this point, these negative emotions may harm the nurses’ physical and emotional health, leading to burnout, missing work, and/or avoidance behavior [9]. For this reason, it is important to offer a balancing organizational intervention, that is, finding the balance point: on the one hand, emphasizing the risks and gravity of the situation, and on the other, assisting in the nurses’ emotional regulation through the provision of appropriate protective equipment, making the nurses feel safer physically and ensuring an inclusive, emotionally supportive environment, promoting their health and mental resilience.

As emotions may also act as beneficial drivers of decision making, influencing judgment and choice [6], an additional way of regulating negative emotions is by strengthening the nurses’ positive emotions, such as a sense of mission and pride. The study’s model demonstrates that, despite the fact that the positive emotions on their own were not directly associated with any of the other study variables, they were associated with the level of negative emotions, so that the stronger the positive emotions were, the lower the negative emotions became. This may imply that even during a health crisis, when negative emotions may be unavoidable, enhancing nursing staffs’ positive emotions may help regulating their emotional distress, better judgment, and choice.

The current study may be subject to several limitations. First, the study is based on a self-reporting method, which may be subject to response bias and selectivity bias. Second, the nonrandomized sampling technique limits the generalizability of our results. Furthermore, the study is a cross-sectional study, conducted only at one point in time during the first wave of the COVID-19 pandemic. Future studies should examine preventive behaviors adopted by nurses and emotional levels over time.

## 5. Conclusions

Policymakers in the healthcare system have a crucial role in managing disaster and crisis situations, such as the COVID-19 pandemic. In order to keep the nursing staff who are at the frontlines of patient care safe, policymakers must choose a policy that takes action to preserve the workers’ physical and emotional health and safety, strengthening workers’ safety and confidence through the use of appropriate protective equipment, evidence-based updated clinical information influencing threat and risk perceptions, and assistance in the regulation of negative emotions and strengthening of positive emotions, such as a sense of mission and professional pride. It is of the utmost importance to find and maintain ways of dealing with the pandemic’s wave-like character (several waves of morbidity with periods of ‘rest’ separating them) to provide a response to nursing staffs’ negative emotions, and to assist them in preserving a balance so that, on the one hand, there is no complacency, and on the other, we avoid a situation where high, intense negative emotions lead to avoidance, burnout, or even emotional harm.

## Figures and Tables

**Table 1:** Participants’ Demographic Characteristics (N = 346).

	Range	
Age (years) M (SD)	21–71	41.49 (10.45)
Nursing experience (years) M (SD)	0–48	16.27 (11.62)
Role N (%)	Registered nurse (RN)	302 (87.3)
	Nursing student	44 (12.7)
Gender N (%)	Female	288 (84.5)
Religion N (%)	Jewish	254 (74.7)
Country of birth N (%)	Israel	238 (70.0)
Marital status N (%)	Married, in an intimate relationship	251 (73.8)
	Single, divorced	89 (26.2)
Economic status N (%)	Below average	89 (27.1)
	Average and above	240 (72.9)
Religiosity level N (%)	Secular	195 (57.4)
	Partly religious	80 (23.5)
	Religious	65 (19.1)
Current main workplace N (%)	Hospital	269 (79.6)
	Other (community service, geriatric facility)	69 (20.4)

Percentages were calculated excluding missing data.

**Table 2:** Means, standard deviations, and intercorrelations for study variables (N = 346).

	M (SD)	2.	3.	4.	5.
1. Negative emotions	2.89 (1.23)	−0.36 ***	0.40 ***	0.32 ***	0.05
2. Positive emotions	3.94 (1.07)		−0.17 **	−0.13 *	0.03
3. Threat perception	5.14 (1.07)			0.44 ***	0.22 ***
4. Risk perception	5.45 (0.80)				0.40 ***
5. Compliance	6.16 (0.86)				

* *p* < 0.05, ** *p* < 0.01, *** *p* < 0.001. Range 1–7.

**Table 3:** Means, standard deviations, and t values for study variables by extent of rule compliance (N = 346).

	Lower Compliance M (SD)	High Compliance M (SD)	t (344)	*p*
Negative emotions	2.87 (1.24)	2.90 (1.22)	0.20	0.844
Positive emotions	3.82 (1.06)	4.03 (1.07)	1.75	0.080
Threat perception	4.88 (1.08)	5.32 (1.02)	3.85	<0.001
Risk perception	5.10 (0.76)	5.68 (0.74)	6.95	<0.001

**Table 4:** Path analysis for nurses’ behavior, threat and risk perceptions, negative and positive emotions (N = 346).

Dependent Variable (R2)	Predictor	β	SE(B)
Threat perception (0.20)	Negative emotions	0.39 ***	0.05
	Positive emotions	−0.05	0.05
Risk perception (0.14)	Negative emotions	0.30 ***	0.05
	Positive emotions	−0.03	0.05
Compliance (0.18)	Negative emotions	−0.08	0.06
	Positive emotions	0.07	0.05
	Threat perception	0.08	0.06
	Risk perception	0.41 **	0.06

** *p* < 0.01, *** *p* < 0.001.

## Data Availability

The data presented in this study are available on request from the corresponding author. The data are not publicly available due the respondents’ privacy.
